# Supplementary material for: The effects of plant-based dietary patterns on the risk of developing gestational diabetes mellitus: A systematic review and meta-analysis
Source: PLoS One. 2023 Oct 4;18(10):e0291732. doi: 10.1371/journal.pone.0291732 (PMC10550137; doi:10.1371/journal.pone.0291732)
Supplement: S1 Appendix — (DOCX) [file pone.0291732.s001.docx]

**S1 Appendix. Search Strategies for Each Database**

| **Database** | **NO.** | **Query** |
| --- | --- | --- |
| PubMed | #1 | "Diet, Vegetarian"[Mesh] |
|  | #2 | plant-based[Title/Abstract] OR plant-based diet[Title/Abstract] OR vegetarian[Title/Abstract] OR vegan[Title/Abstract] OR vegetable[Title/Abstract] OR dietary fiber[Title/Abstract] OR dietary pattern[Title/Abstract] OR food pattern[Title/Abstract] |
|  | #3 | "Diabetes, Gestational"[Mesh] |
|  | #4 | gestational diabetes mellitus[Title/Abstract] OR gestational diabetes[Title/Abstract] OR GDM[Title/Abstract] |
|  | #5 | #1 or #2 |
|  | #6 | #3 or #4 |
|  | #7 | #5 and #6 |
| Web of Science | #1 | TS= (plant-based OR plant-based diet OR vegetarian OR vegan OR vegetable OR dietary fiber OR dietary pattern OR food pattern) |
|  | #2 | TS= (gestational diabetes mellitus OR gestational diabetes OR GDM) |
|  | #3 | #1 and #2 |
| Embase | #1 | 'pregnancy diabetes mellitus'/exp |
|  | #2 | 'gestational diabetes mellitus' OR 'gestational diabetes' OR 'GDM' |
|  | #3 | 'vegetarian diet'/exp |
|  | #4 | 'plant-based' OR 'plant-based diet' OR 'vegetarian' OR 'vegan' OR 'vegetable' OR 'dietary fiber' OR 'dietary pattern' OR 'food pattern' |
| CNKI | SU=('膳食模式'+'膳食结构'+'饮食模式' AND SU=('妊娠期糖尿病'+'妊娠糖尿病'+'GDM') | |
|  | Translated to English:  SU=('dietary pattern'+'dietary structure'+'food pattern' AND SU=('gestational diabetes mellitus '+'gestational diabetes'+'GDM') | |
| Wangfang | 题名或关键词:(膳食模式 or 膳食结构 or 饮食模式) and 题名或关键词:( 妊娠期糖尿病 or 妊娠糖尿病 or GDM) | |
|  | Translated to English:  Title or Keywords: (dietary pattern or dietary structure or food pattern) and Title or Keywords:(gestational diabetes mellitus or gestational diabetes or GDM) | |
| VIP | M=(膳食模式 OR 膳食结构 OR 饮食模式) AND M=(妊娠期糖尿病 OR 妊娠糖尿病 OR GDM) | |
|  | Translated to English:  M=(dietary pattern OR dietary structure OR food pattern) AND M=( gestational diabetes mellitus OR gestational diabetes OR GDM) | |
