# Supplementary material for: The effects of plant-based dietary patterns on the risk of developing gestational diabetes mellitus: A systematic review and meta-analysis
Source: PLoS One. 2023 Oct 4;18(10):e0291732. doi: 10.1371/journal.pone.0291732 (PMC10550137; doi:10.1371/journal.pone.0291732)
Supplement: S2 Appendix — (DOCX) [file pone.0291732.s002.docx]

**S2 Appendix. Assessment of Individual Study Bias.**

| **Study ID** | **SELECTION** | | | | **COMPARABILITY** | **OUTCOME** | | | **Total score** |
| --- | --- | --- | --- | --- | --- | --- | --- | --- | --- |
|  | Representativeness of the Exposed Cohort | Selection of the Non-Exposed Cohort | Ascertainment of Exposure | Demonstration That Outcome of Interest Was Not Present at Start of Study | Comparability of Cohorts on the Basis of the Design or Analysis | Assessment of Outcome | Was Follow-Up Long Enough for Outcomes to Occur | Adequacy of Follow-Up of Cohorts |  |
| Chen 2021 | * | * | * | * | * | None | * | * | 7 |
| Wang 2021 | * | * | * | * | ** | * | * | * | 9 |
| Yisahak 2021 | * | * | * | * | * | * | * | * | 8 |
| Mahendra 2022 | * | * | * | * | * | * | * | * | 8 |
| Schoenaker 2015 | * | * | * | * | * | None | * | * | 7 |
| Zhou 2018 | * | * | * | * | ** | * | * | * | 9 |
| Mak 2018 | * | * | * | * | * | * | * | * | 8 |
| He 2015 | * | * | * | * | * | * | * | * | 8 |
| Yong 2020 | * | * | * | * | * | * | * | * | 8 |
| Wang 2021 | * | * | * | * | ** | * | * | * | 9 |
